# Acupuncture for Post-stroke Shoulder-Hand Syndrome: A Systematic Review and Meta-Analysis

**DOI:** 10.3389/fneur.2019.00433

**Published:** 2019-04-26

**Authors:** Shaonan Liu, Claire Shuiqing Zhang, Yiyi Cai, Xinfeng Guo, Anthony Lin Zhang, Charlie Changli Xue, Chuanjian Lu

**Affiliations:** ^1^The Second Affiliated Hospital of Guangzhou University of Chinese Medicine, Guangzhou, China; ^2^The Second Clinical College of Guangzhou University of Chinese Medicine, Guangzhou, China; ^3^Guangdong Provincial Hospital of Chinese Medicine, Guangzhou, China; ^4^Guangdong Provincial Academy of Chinese Medical Sciences, Guangzhou, China; ^5^China–Australia International Research Centre for Chinese Medicine, School of Health and Biomedical Sciences, RMIT University, Melbourne, VIC, Australia

**Keywords:** acupuncture, stroke, shoulder-hand syndrome, rehabilitation, systematic review

## Abstract

**Background:** Shoulder-hand syndrome (SHS) is prevalent in hemiplegic patients after stroke. Potential benefits of acupuncture were shown in recent clinical trials. This systematic review aimed to comprehensively evaluate the safety and efficacy of acupuncture for SHS in stroke patients.

**Methods:** Five English databases (PubMed, Embase, CINAHL, CENTRAL, and AMED) and four Chinese databases (CBM, CNKI, CQVIP, and Wanfang) were searched from their inceptions to January 2019. Randomized, controlled trials that evaluated the add-on effects of acupuncture to rehabilitation for post-stroke SHS were identified.

**Results:** Thirty-eight studies involving 3,184 participants fulfilled the eligible criteria and were included in the review. The overall meta-analysis showed that acupuncture combined with rehabilitation significantly improved motor function (upper-limb Fugl-Meyer Assessment (FMA): 34 studies, mean difference (MD) 8.01, 95% confidence interval (CI) [6.69,9.33]), and reduced pain (visual analog scale (VAS): 25 studies, MD −1.59, 95%CI [−1.86,−1.32]). It also improved activities of daily living (ADL) when compared with rehabilitation alone (ADL: 11 studies, MD 9.99, 95%CI [5.91,14.06]). However, the certainty of evidence of all these outcomes was assessed as “low.” Subgroup analyses of acupuncture stimulation types and treatment duration all showed significant add-on effects comparing with rehabilitation alone. The safety of acupuncture was unclear because there is a lack of detailed reporting of adverse events in most of the included studies.

**Conclusions:** Acupuncture therapy seems effective for motor function, pain relief and activities of daily living in stroke patients with mild SHS, when it is used in combination with rehabilitation. The low certainty of evidence downgrades our confidence in making recommendations to clinical practice.

## Introduction

Shoulder-hand syndrome (SHS) is a common condition among people who have had a stroke, with its reported prevalence ranging from 12% to 49% (1, 2). The main symptoms of SHS include pain, hyperalgesia, joint swelling and limitations in range of motion (ROM) (3). Post-stroke SHS is also named type I complex regional pain syndrome (CRPS) or reflex sympathetic dystrophy (4). The key to effectively treating SHS is believed to be an expert multidisciplinary team that provides individualized therapy (5). There is a wide range of treatment options available to help manage post-stroke SHS, including physical therapy, medications, regional anesthesia techniques and neuromodulation. However, there is insufficient evidence to support their efficacy (5).

Acupuncture, one of the most popular traditional Chinese medicine therapies, has been widely used in the clinical management of stroke (6). Several systematic reviews have assessed its efficacy for improving stroke rehabilitation using outcomes in motor function recovery and disability, but results are inconsistent (7–11). Three reviews published before 2010 showed acupuncture did not improve motor function or dependency outcomes after rehabilitation (8, 9, 11). However, two more recently published reviews suggested acupuncture might aid rehabilitation in several areas, including motor function recovery and pain relief (7, 10).

Three systematic reviews specifically evaluating acupuncture for post-stroke SHS have been published (12–14). Two of these were published before 2013 (12, 13), so they don't include recently published clinical evidence. Moreover, one review including three studies did not perform quantitative synthesis due to clinical heterogeneity (13). The third review (14) does not evaluate the effectiveness of electro-acupuncture, and only two of its included studies evaluated the effectiveness of acupuncture combined with routine care. Considering electro-acupuncture is commonly used in the clinical management of stroke complications, the implication of the review results (14) for clinical practice is limited. Therefore, we conducted this systematic review looking at the most recent evidence of acupuncture (including electro-acupuncture) as an additional therapy in the clinical management of post-stroke SHS.

## Methods

### Study Design

This systematic review included randomized controlled trials (RCT) or quasi-RCTs that were published in English or Chinese and evaluated acupuncture's effects as an additional therapy for post-stroke SHS. Quasi-RCTs were evaluated using the same methods applied to RCTs.

We registered its protocol with the PROSPERO international prospective register of systematic reviews (CRD 42016050446).

### Participants

We limited participants to people who were diagnosed with post-stroke SHS. The stroke (ischemic or hemorrhagic) diagnosis needed to be confirmed by computer tomography or magnetic resonance imaging. The SHS or CRPS type I diagnosis was based on clinical symptoms, including pain, motor disturbances and skin changes (3).

### Intervention

We included RCTs or quasi-RCTs that evaluated the effects of manual or electro-acupuncture combined with routine care or rehabilitation as the experimental intervention in this review. Studies that assessed auricular acupuncture or other types of recently developed acupuncture forms, such as floating acupuncture, were excluded from this review.

The rehabilitation therapy used in RCTs or quasi-RCTs could be a combination of physiotherapy and occupational therapy, such as active ROM, passive ROM, mirror visual feedback, Bobath therapy, alternating heat and cold baths, and massage. The comparator was the same as the rehabilitation therapy used in the intervention group, but without acupuncture. Studies that used placebo or sham acupuncture and the same rehabilitation therapy in the control group were also eligible for inclusion.

### Outcome

RCTs or quasi-RCTs that reported validated SHS outcome measures as below were considered for inclusion in this review.

Primary outcome measures: (1) motor function: Fugl-Meyer Assessment (FMA) upper limb; (2) pain assessment using visual analog scale (VAS) or numerical rating scale (NRS).

Secondary outcome measures: (1) Barthel Index (BI) or Modified Barthel Index (MBI), which is assessed for self-care and activities of daily living; (2) ROM; and (3) adverse events.

### Literature Search

We searched nine databases, five in English, and four in Chinese languages, from their inceptions to January 2019. They are: PubMed, Embase, Cumulative Index of Nursing and Allied Health Literature (CINAHL), Cochrane Central Register of Controlled Trials (CENTRAL), the Allied and Complementary Medicine Database (AMED), China BioMedical Literature (CBM), China National Knowledge Infrastructure (CNKI), Chonqing VIP (CQVIP), and Wanfang. We also searched clinical trial registration agencies, including the Chinese Clinical Trial Registry and National Institutes of Health Register (Clinical Trials.gov), and hand-searched references lists of included studies and relative systematic reviews. The search terms and search strategy we used are presented in Appendix S1.

### Data Extraction

Two researchers (SL, YC) independently screened the titles, abstracts and full text of studies to assess eligibility. Any uncertainty was resolved through discussion with CZ. SL and YC developed a standardized data extraction file using Epidata software 3.1 (The EpiData Association, Odense, Denmark, 2003–2008). They also extracted data independently and cross-checked it for accuracy. Extracted data included: author, publication year, diagnostic criteria, duration of disease, sample size, participants' age, details of interventions and all clinical outcomes.

### Risk of Bias and Certainty of Evidence Assessment

Two researchers (SL, YC) used the Cochrane risk of bias tool to assess the methodological quality of the included studies. They assessed seven items: sequence generation, allocation concealment, blinding of participants and personnel, blinding of outcome assessors, incomplete outcome data, selective reporting, and other sources of bias. The studies were judged as having a “low,” “high,” or “unclear” risk of bias. SL and YC resolved any difference in their assessment of a study by discussing it with a third researcher (CZ).

The Grading of Recommendations Assessment, Development and Evaluation Approach (GRADE) was used to evaluate the certainty of evidence. Five items involving risk of bias, inconsistency, imprecision, indirectness and publication bias were investigated for the clinical important outcomes.

### Data Analysis

The included studies calculated acupuncture's safety and efficacy when combined with rehabilitation to treat SHS, compared to rehabilitation alone. Data analyses were performed in a random-effects model using the RevMan software (version 5.3). Dichotomous data was reported as relative risk (RR) with corresponding 95% confidence intervals (CI). For continuous data, the mean difference (MD) with 95% CI was calculated.

The following subgroup analyses were planned: (1) subgroups of manual or electro-acupuncture; (2) subgroups of treatment duration ≤4 or >4 weeks. Since an inappropriate randomization sequence generation was associated with biased intervention effects (15), we conducted a sensitivity analysis to check the robustness of the results by only including the RCTs that were assessed as “low” risk of bias for this item.

## Results

### Descriptions of Studies

**Figure 1:**
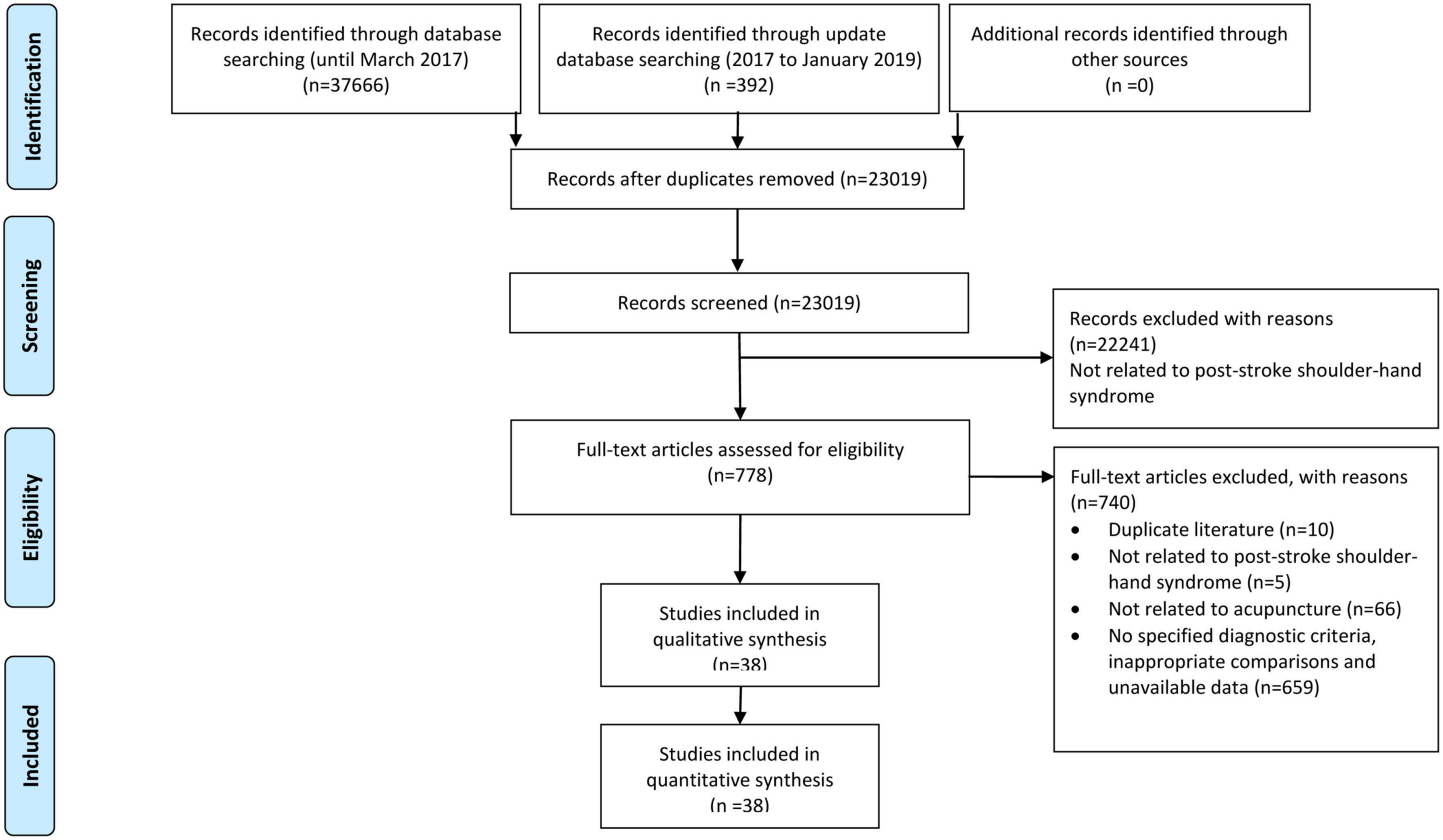
PRISMA flow chart.

We identified 36 eligible studies (16–51) by searching Chinese and English databases up to March 2017. An updated search, including studies from 2017 to January 2019, located another two studies that met the inclusion criteria (52, 53). As a result, 38 RCTs involving 3,184 participants were included in this review (Figure 1).

As reported by the included studies, SHS occurred after ischemic stroke in four studies (40, 42, 44, 45), and after hemorrhagic or ischemic stroke in 23 studies. The remaining 11 studies (17–19, 23, 28, 30, 31, 41, 46, 47, 50) did not include information about the type of stroke. The duration of stroke ranged from 10 days (46) to 19 months (17). The severity of SHS was reported by 27 studies and divided into acute-hyperaemic (stage I), dystrophic-ischaemic (stage II) and atrophic (stage III) according to clinical symptoms (54). Twenty studies recruited participants at stage I with mild symptoms of <3 months of SHS. The remaining seven studies included participants with stage II or III.

All of the eligible trials compared body acupuncture plus routine rehabilitation with routine rehabilitation alone. Of all, 12 studies also applied electrical stimulation with acupuncture (16–18, 20, 21, 32, 34, 36–39, 46). The most commonly used acupuncture points were LI15 *Jianyu*, LI11 *Quchi*, TE5 *Waiguan*, LI4 *Hegu*, TE14 *Jianliao*, LI10 *Shousanli*, HT1 *Jiquan*, PC6 *Neiguan*, and SI9 *Jianzhen*. Routine rehabilitation included active ROM, passive ROM, mirror visual feedback, Bobath therapy, alternating heat and cold baths, and massage. Treatment duration ranged from 2 to 8 weeks. None of the included studies involved a follow-up phase to investigate acupuncture's long-term effects.

Thirty-four studies used FMA to evaluate acupuncture's effects on upper limb motor function. Pain severity was assessed with VAS in 26 studies. None of the included studies reported NRS. For secondary outcomes, 11 studies reported BI or MBI, six studies reported ROM, and five studies reported adverse events (Table 1).

**Table 1:** Basic characteristics.

**References**	**Sample size**	**Stroke type**	**SHS severity**	**Duration of condition**	**First stroke onset**	**Intervention**	**Comparisons**	**Acupoints**	**Acupuncture treatment details**	**Treatment duration**	**Outcome measures**
Chen (32)	96/85	Cerebral infarction and hemorrhage	NS	NS	NS	EA + Rehab	Rehab	EX-B2, GB21, SI11, LI15, TE14, SI9, LI14, PC3, LU5, TE5, SI3, LI4, HT1	Once/d, 6 d/w	4 w	FMA
Chen et al. (51)	48/46	Cerebral infarction and hemorrhage	Stage I	I: 34.91 d; C: 35.41 d	Yes	Acupuncture + Rehab	Rehab	LI15, TE14, Jianqian, SI9, LI11, TE5, LI10, EX-UE9	30 min, once/d, 6 d/w	4 w	FMA, VAS, BI
Duan et al. (17)	60/60	NS	NS	14 d−19 m	NS	EA + Rehab	Rehab	SI11, SI9, SI10, LU2, LI11, LI10, HT3, Jianqian, Jianfeng, Ashi point	20 min, once/d, 5 d/w	8 w	FMA
Feng and Ma (50)	69/57	NS	Stage I	33.1 d	NS	Acupuncture + Rehab	Rehab	PC6, LI5, ST9, LU5, HT1	30 min, once/d	4 w	FMA, VAS, ROM
Gao et al. [2011^16^]	16/16	Cerebral infarction and hemorrhage	NS	48.5 d	NS	EA + Rehab	Rehab	LI15, TE14, LI14, LI11, TE5, TE4, KI15, EX-UE9	30 min, once/d	4 w	VAS, BI
He and Gao (49)	60/60	Cerebral infarction & hemorrhage	Stage I	I: 21.07 d; C: 20.32 d	NS	Acupuncture + Rehab	Rehab	CV12, CV4, ST24, ST26, Shangfengshidian, Shengfengshiwaidian	30 min, once/d	3 w	FMA, VAS, MBI
Hou (20)	30/30	Cerebral infarction and hemorrhage	Stage I	I: 29.8 d; C: 31.5 d	NS	EA + Rehab	Rehab	GV20, GV24, EX-HN3, LI11, TE5, LI4	50–100 Hz, 30 min, once/d, 5 d/w	4 w	FMA, VAS
Huang et al. (48)	30/30	Cerebral infarction and hemorrhage	Stage I	I: 46.8 d; C: 45.4 d	Yes	Acupuncture + Rehab	Rehab	EX-UE9, EX-UE8	20 min, once/d, 5 d/w	3 w	FMA
Jia et al. (21)	28/24	Cerebral infarction and hemorrhage	Stage I	I: 28.5 d; C: 31 d	NS	EA + Rehab	Rehab	GV20, GV24, EX-HN3, LI15, TE14, LI11, TE5, LI4	50–100 Hz, 30 min, once/d, 5 d/w	4 w	FMA, VAS
Li (18)	60/60	NS	NS	NS	NS	EA + Rehab	Rehab	LI1, LI2, LI3, LI5, LI11,	2–30 Hz, 15 min, once/d	14 d	FMA
Li and Tu (34)	45/45	Cerebral infarction and hemorrhage	Stage I	I: 4.29 m; C: 3.93 m	Yes	EA + Rehab	Rehab	LI10, LI15, LI15, LI11, PC6, TE5, LI4, Ashi point	50–100 Hz, 30 min, once/d, 5 d/w	6 w	FMA, VAS, MBI
Li et al. (47)	46/46	NS	NS	NS	NS	Acupuncture + Rehab	Rehab	LU9, ST36, LI11, LI10, LI15, GB39, BL62	30 min, once/d, 6 d/w	1 m	FMA
Liang and Liu (46)	42/42	NS	Stage I, II	I: 10 d−3 m; C: 15 d−3 m	NS	EA + Rehab	Rehab	GB21, LI15, SI9, TE14, LI14, SI11, LI11, TE5, SI3, PC8, LI4	30 min, once/ d	30 d	FMA, VAS
Liao (33)	45/45	Cerebral infarction and hemorrhage	NS	I: 48.6 d; C: 50.7 d	NS	Acupuncture + Rehab	Rehab	GV26, PC6, HT5, HT1, ST36, GB39, SP6,LI15, LI4, LI11, TE13, TE9	30 min, once/d	5 w	FMA, VAS, MBI
Lin et al. (30)	40/40	NS	Stage I	I: 29.3 d; C: 28.6 d	NS	Acupuncture + Rehab	Rehab	Piantan, Jiantong, Shengti, Jiansanzhen, Ashi point, HT1, PC6, LU5	30 min, once/d	4 w	FMA, VAS
Liu (23)	40/40	NS	NS	I: 36.25 d; C: 37.25 d	NS	Acupuncture + Rehab	Rehab	Jiantong	30 min, once/d, 5 d/w	40 d	FMA, VAS, ROM
Liu and Zhang (52)	49/49	Cerebral infarction and hemorrhage	NS	I: 48.18 d; C: 47.18 d	Yes	Acupuncture + Rehab	Rehab	LI15, LI11, TE5, LI4, SI3, TE3	20 min, once/d, 6 d/w	4 w	FMA, VAS
Qin et al. (27)	40/40	Cerebral infarction and hemorrhage	Stage I	I: 4 m; C: 4 m	Yes	Acupuncture + Rehab	Rehab	Four points in head	30 min, once/d, 6 d/w	NS	VAS
Shang et al. (28)	40/40	NS	Stage I, II	I: 5.23 m; C: 5.02 m	NS	Acupuncture + Rehab	Rehab	LI15, Jianqian, TE14, HT1, LI14, LI11, PC6, LI4	40 min,2 times/d	30 d	FMA, ROM
Su et al. (24)	22/21	Cerebral infarction and hemorrhage	Stage I, II	I: 44.2 d; C: 43.7 d	NS	Acupuncture + Rehab	Rehab	Piantan, Jiantong, Shengti	once/d	2 w	FMA
Tang et al. (45)	30/30	Cerebral infarction	Stage I	I: 48.54 d; C: 49.12 d	NS	Acupuncture + Rehab	Rehab	SI11, LI15, LI14, Jianqian, KI15, SI3	40 min, 6 d/w	28 d	FMA, VAS
Wan et al. (25)	40/40	Cerebral infarction and hemorrhage	Stage I, II	I: 24.53 d; C: 24.4 d	NS	Acupuncture + Rehab	Rehab	Piantan, Jiantong, Bitong, Wantong	once/d, 5 d/w	4 w	FMA, MBI
Wan et al. (29)	60/60	Cerebral infarction and hemorrhage	Stage I, II	I: 38.4 d; C: 33.0 d	NS	Acupuncture + Rehab	Rehab	LU9, ST36, GB39, TE5, LI10, LI11, LI15	30 min, once/d	28 d	FMA
Wang et al. (31)	31/31	NS	NS	I: 10–26 d; C: 12–29 d	NS	Acupuncture + Rehab	Rehab	Ashi point, LI15, Jianqian, SI9, HT1, LU5, LI10, LI11, PC6, TE5, LI4	30 min, once/d, 5 d/w	5 w	MBI
Wang et al. (53)	71/71	Cerebral infarction and hemorrhage	StageI, II, III	I: 66.8 d; C: 67.4 d	NS	Acupuncture + Rehab	Rehab	LI15, TE14, GB21, SI9, LI17, LI11, TE5, LI4, SI6, KI15, CV6, ST36	15 min, once/d	21 d	FMA, MBI
Wu (39)	30/30	Cerebral infarction and hemorrhage	Stage I	15 d−3 m	NS	EA + Rehab	Rehab	EX-B2, TE14, TE13, LI15, LI14, SI9, HT1, LI4, LI11, KI15, TE5, SI6, SI3	30 min, once/d, 6 d/w	4 w	ROM
Xie (40)	30/30	Cerebral infarction	NS	15 d−3 m	NS	Acupuncture + Rehab	Rehab	Tousanzhen, Jiansanzhen, HT1, LU5, PC6	once/d, 5 d/w	4 w	FMA, VAS, AE
Xu (36)	36/36	Cerebral infarction and hemorrhage	Stage I	I: 5–44 d; C: 4–47 d	NS	EA + Rehab	Rehab	LI1, LI2, LI3, LI5, LI11	30 min, once/d, 6 d/w	4 w	FMA, VAS, AE
Xu et al. (19)	42/40	NS	Stage I	I: 33.45 d; C: 27.14 d	NS	Acupuncture + Rehab	Rehab	Jiansanzhen, HT1, LU5, PC6	30 min, once/d, 6 d/w	5 w	VAS, FMA, ROM, AE
Xu et al. (44)	40/40	Cerebral infarction	Stage I	I: 47.2 d; C: 48.6 d	Yes	Acupuncture + Rehab	Rehab	LI15, LI14, HT1, Jianqian, Jianhou, KI15, SI3	40 min	14 d	FMA, VAS
Yang et al. (43)	40/40	Cerebral infarction and hemorrhage	Stage I	I: 87.93 d; C: 95.8 d	NS	Acupuncture + Rehab	Rehab	LI15, LI11, LI10, PC6, LI4	30 min, once/d	20 d	FMA, VAS
Yin et al. (26)	30/30	Cerebral infarction and hemorrhage	Stage I	I: 67.17 d; C: 67.10 d	NS	Acupuncture + Rehab	Rehab	Six meridians on hand, including LI15, LI11, TE14, GB21, etc.	20–30 min, once/d, 6 d/w	4 w	FMA, VAS
You (37)	31/31	Cerebral infarction and hemorrhage	Stage I, II, III	I: 31.89 d; C: 33.45 d	NS	EA + Rehab	Rehab	LI15, TE14, SI9, LI14, SI11, LI10, TE5, LI4, EX-UE9	30 min, once/d, 6 d/w	4 w	FMA, VAS, MBI, ROM
Zhang (38)	30/30	Cerebral infarction and hemorrhage	Stage I	I: 58.64 d; C: 54.97 d	NS	EA + Rehab	Rehab	LI15, LI11, LI4, TE5, LI10	30 min, once/d, 5 d/w	3 w	FMA, VAS, AE
Zhao (42)	24/23	Cerebral infarction	Stage I	I: 77.67 d; C: 65.65 d	Yes	Acupuncture + Rehab	Rehab	TE14, SI9, Jianqian, LI15, Ashi point, LI11, LI5, TE5	30 min, once/d, 6 d/w	28 d	FMA, VAS, MBI, AE
Zheng et al. (35)	39/38	Cerebral infarction and hemorrhage	Stage I	I: 41.72 d; C: 42.48 d	NS	Acupuncture + Rehab	Rehab	LI15, TE14, SI9, LI11, LI10, TE5, LI4, SI3	30 min, once/d, 5 d/w	4 w	FMA, VAS, BI
Zhong et al. (41)	30/30	NS	Stage I	I: 28.4 d; C: 29.2 d	NS	Acupuncture + Rehab	Rehab	Piantan, Jiantong, Shengti, Jiansanzhen, Ashi point, HT1, PC6, LU5	30 min, once/d	30 d	FMA, VAS
Zhou et al. (22)	45/45	Cerebral infarction and hemorrhage	NS	I: 55.13 d; C: 57.08 d	NS	Acupuncture + Rehab	Rehab	LI4, LU7, LI11, LI15, CV21, CV4	30 min, once/d, 6 d/w	1 m	FMA, VAS

*All included articles were published in Chinese journals. AE, adverse events; BI, Barthel Index; C, comparison; d, day; EA, electroacupuncture; FMA, Fugl-Meyer Assessment (upper limb); I, intervention; m, month; MBI, Modified Barthel Index; NS, not specified; Rehab, rehabilitation; ROM, shoulder range of motion; VAS, visual analog scale; w, week; SHS, shoulder-hand syndrome*.

### Risk of Bias of Included Studies

**Figure 2:**
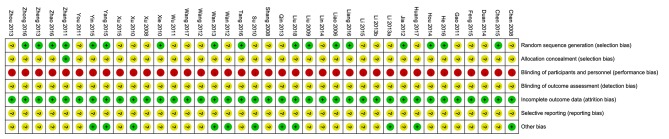
Risk of bias of each study.

Seventeen studies (45%) were assessed as “low” risk of bias for sequence generation, and the remaining studies were assessed as “unclear” risk due to a lack of information. Only one study (38) used opaque envelopes to conceal the allocation procedure, so it was assessed as “low” risk of bias for this item. Blinding of participants and acupuncturists was not performed in any study. Blinding of outcome assessors was assessed as “unclear” risk of bias for all studies due to a lack of information. We also assessed selective outcome reporting as “unclear” risk of bias for all studies, because none of the studies had published their protocols. For incomplete outcome data, we assessed all studies as “low” risk of bias because there was no missing outcome data. Eleven studies (18, 19, 24–27, 29, 32, 43, 48, 52) reported the funding source and showed balanced baseline data, so we assessed the relative other bias as “low” risk. Details are summarized in Figure 2 and Figure S1.

### Acupuncture's Effects on Post-stroke SHS

#### FMA (Upper Limb)

Thirty-four studies reported data on upper limb FMA scores when acupuncture was combined with rehabilitation compared to rehabilitation alone. Meta-analysis of 29 studies showed the combination therapy had a significant superior effect (MD: 8.01, 95% CI [6.69, 9.33]; *I*^2^ = 78%). We did not include the remaining five studies (17, 18, 32, 33, 48) in our analysis due to their inappropriate data reporting: three studies (18, 33, 48) only reported scores of certain items without the total score, one study (32) reported FMA as categorical data, and one study (17) did not report FMA data for the control group.

#### VAS

Compared with routine rehabilitation alone, acupuncture combined with routine rehabilitation showed superior effects on VAS scores in 25 studies (MD: −1.59, 95% CI [−1.86, −1.32]; *I*^2^ = 87%). We excluded one study (27) in the meta-analysis because its data was reported with median and interquartile ranges.

#### ADL

Eleven studies reported acupuncture's effects on ADL performance, as measured by the Barthel Index (BI) or Modified Barthel Index (MBI). Meta-analysis showed that acupuncture combined with routine rehabilitation enhanced ADL performance more than rehabilitation alone did (MD: 9.99, 95% CI [5.91, 14.06]; *I*^2^ = 86%).

#### ROM

Acupuncture combined with routine rehabilitation improved shoulder abduction by an average of 11.94 degrees (three studies (19, 23, 37): 95% CI [9.44, 14.45]; *I*^2^ = 0%), shoulder internal rotation by 18.72 degrees (one study (37): 95% CI [9.63, 27.81]), and shoulder external rotation by 15.73 degrees (one study (37): 95% CI [6.82, 24.64]), when compared with rehabilitation alone.

#### Subgroup Analysis and Sensitivity Analysis

**Figure 3:**
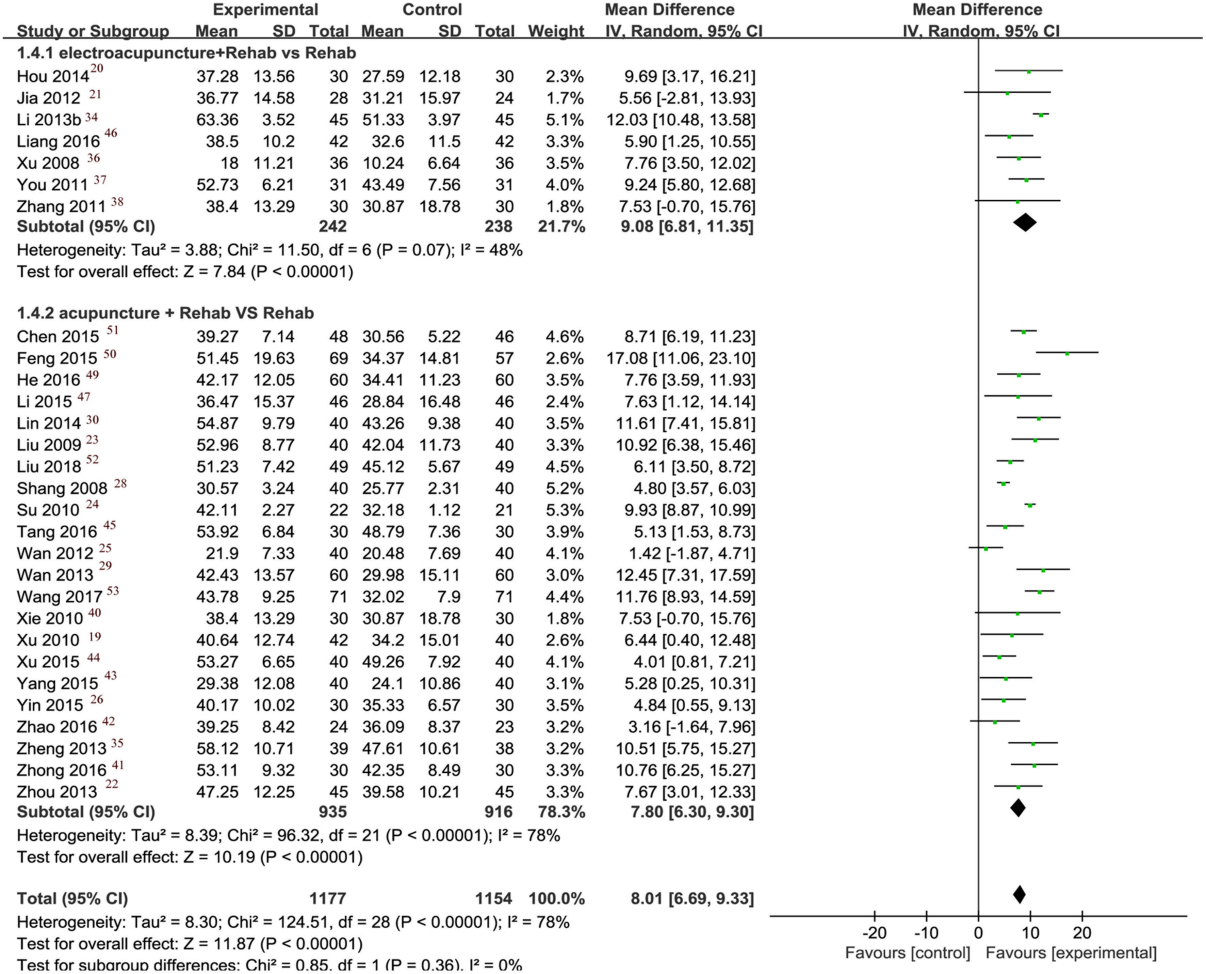
Forest plot of FMA.

**Figure 4:**
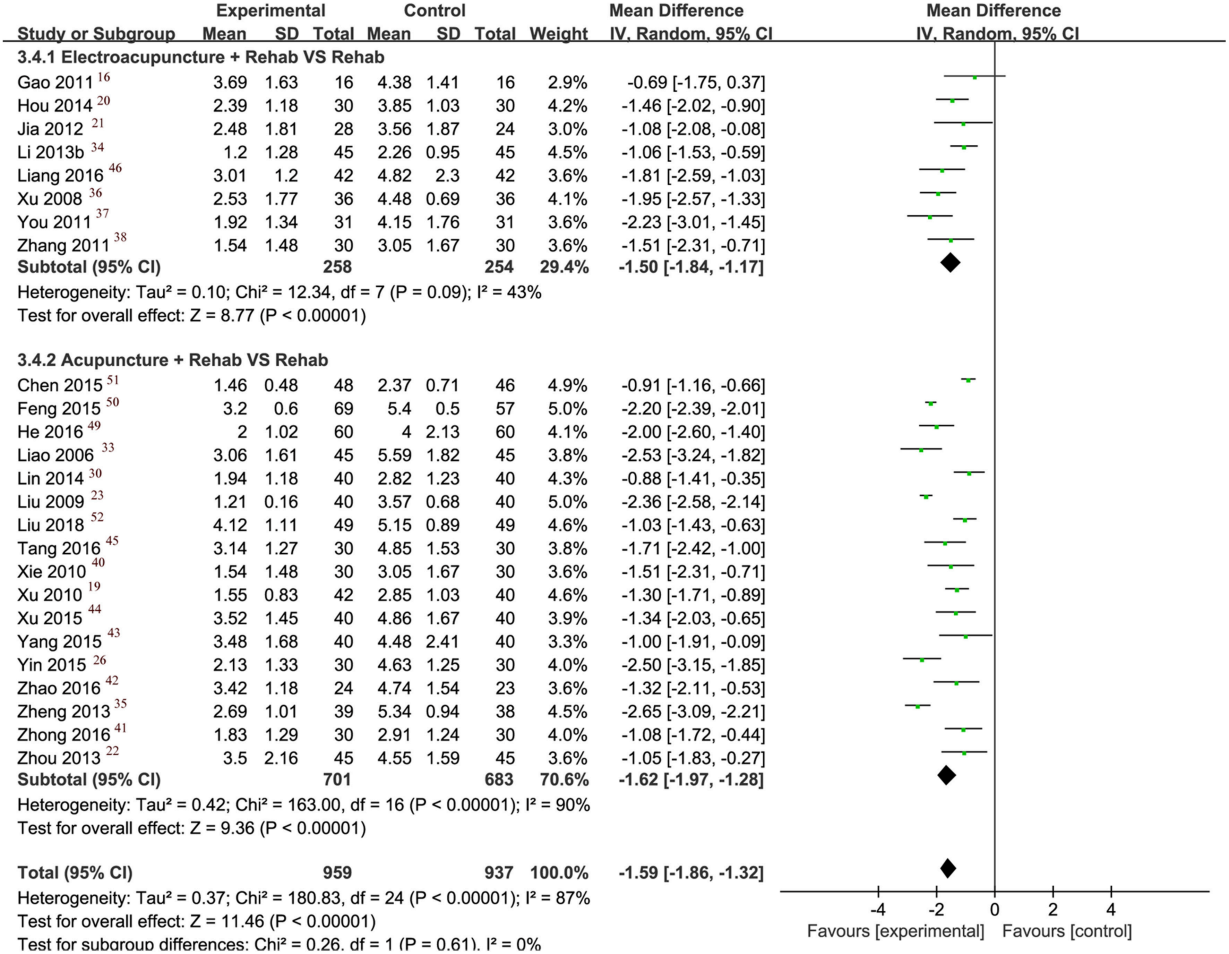
Forest plot of VAS.

Subgroup analysis was conducted to examine whether adding electrical stimulation to manual acupuncture will affect primary outcomes. Manual and electro-acupuncture combined with routine rehabilitation improved FMA and pain VAS outcomes more than rehabilitation alone did (Figures 3, 4). The effect of electro-acupuncture (MD 9.08, 95% CI [6.81, 11.35]) seems greater in magnitude than manual acupuncture (MD 7.80, 95% CI [6.30, 9.30]) for improving upper limb motor function, however, there was not any direct analysis to compare electro-acupuncture with manual acupuncture in this research.

Further subgroup analyses in the manual or electro-acupuncture group looked at the impact of treatment duration on outcomes. We found that treatment duration (≤4 weeks or >4 weeks) did not significantly change the outcome. However, the number of studies included in each subgroup was relatively small (Table 2).

**Table 2:** Subgroup analysis and sensitivity analysis.

**Analysis**	**FMA (upper limb) (number of studies, number of participants in intervention/control group, mean difference, 95% CI, *I*^**2**^)**	**VAS (number of studies, number of participants in intervention/control group, mean difference, 95% CI, *I*^**2**^)**
**Acupuncture** **+** **rehabilitation vs. rehabilitation**	22, 935/916, 7.80 [6.30, 9.30], 78%	17, 701/683, −1.62 [−1.97, −1.28], 90%
Treatment duration ≤ 4 weeks^a^	16, 692/675, 7.79 [6.02, 9.57], 77%	12, 499/483, −1.60 [−2.02, −1.17], 90%
Treatment duration > 4 weeks^a^	6, 243/241, 7.74 [5.04, 10.43], 62%	5, 202/200, −1.68 [−2.34, −1.03], 89%
Reported appropriate randomized allocation methods^b^	12, 480/476, 7.64 [6.09, 9.18], 40%	12, 465/461, −1.73 [−2.18, −1.27], 91%
**Electroacupuncture** **+** **rehabilitation vs. rehabilitation**	7, 242/238, 9.08 [6.81, 11.35], 48%	8, 258/254, −1.50 [−1.84, −1.17], 43%
Treatment duration ≤ 4 weeks^a^	5, 155/151, 8.46 [6.18, 10.75], 0%	6, 171/167, −1.57 [−1.97, −1.18], 37%
Treatment duration > 4 weeks^a^	2, 87/87, 9.37 [3.42, 15.33], 83%	2, 87/87, −1.37 [−2.09, −0.64], 61%
Reported appropriate randomized allocation methods^b^	4, 130/126, 7.00 [3.81, 10.18], 0%	4, 130/126, −1.50 [−1.87, −1.13], 0%

a Subgroup analysis.

b Sensitivity analysis.

*FMA, Fugl-Meyer Assessment; VAS, Visual Analog Scale*.

Sensitivity analysis was performed by only including studies that reported appropriate randomization sequence generation methods. The results were similar to that of the overall pool, with lower heterogeneity. The results also showed that acupuncture combined with routine rehabilitation produces better outcomes for FMA and VAS than rehabilitation alone (Table 2).

#### Publication Bias

Publication bias was evaluated based on FMA and VAS, which were reported in 29 and 25 studies, respectively. Egger's test demonstrated there was no significant publication bias (FMA: *t* = −0.39, *P* = 0.702; VAS: *t* = 1.50, *P* = 0.147).

#### Adverse Events

Thirty-three studies did not mention adverse events. Four studies reported that no adverse event occurred during the trials (19, 36, 40, 42). One RCT (38) described that three participants in the intervention group and two in the control group reported bruising after treatment. These events were considered to be mild and did not require any medical management. The study did not explore the causality of these adverse events (38).

#### Assessment of Evidence

The evidence of all these outcomes was assessed as “low” certainty. The certainty of evidence was downgraded mainly due to the limitations in study design and inconsistency of results. The summary of finding table was presented in Table 3.

**Table 3:** Certainty of evidence (GRADE).

**Certainty assessment**							**№ of patients**		**Effect**		**Certainty**	**Importance**
**№ of studies**	**Study design**	**Risk of bias**	**Inconsistency**	**Indirectness**	**Imprecision**	**Other considerations**	**Acupuncture + Rehab**	**Rehab**	**Relative (95% CI)**	**Absolute risk difference between two groups (95% CI)**		
**FMA UPPER LIMB**												
29	Randomized trials	Serious^a^	Serious^b^	Not serious	Not serious	None	1,177	1,154	–	MD **8.01 higher** (6.69 higher to 9.33 higher)	⊕⊕○○ LOW	Critical
**VAS**												
25	Randomized trials	Serious^a^	Serious^b^	Not serious	Not serious	None	959	937	–	MD **1.59 lower** (1.86 lower to 1.32 lower)	⊕⊕○○ LOW	Critical
**ADL**												
11	Randomized trials	Serious^a^	Serious^b^	Not serious	Not serious	None	450	446	–	MD **9.99 higher** (5.91 higher to 14.06 higher)	⊕⊕○○ LOW	Important
**ROM Abduction**												
3	Randomized trials	Serious^a^	Not serious	Not serious	Serious^c^	None	113	111	–	MD **11.94 degree higher** (9.44 higher to 14.45 higher)	⊕⊕○○ LOW	Important

a Lack of blinding of participants and personnel.

b Substantial statistical heterogeneity.

c Small sample size limits certainty of results.

*FMA, Fugl-Meyer Assessment; VAS, visual analog scale; ADL, activities of daily living; ROM, range of motion; Rehab, rehabilitation; CI, Confidence interval; MD, Mean difference; GRADE, The Grading of Recommendations Assessment, Development and Evaluation*.

## Discussion

In this review, we identified 38 RCTs that evaluated the effects of adding acupuncture to routine rehabilitation to treat post-stroke SHS. Results showed that the combination of acupuncture and routine rehabilitation was superior to rehabilitation alone for improving motor function and ADL, and reducing pain. Manual and electro-acupuncture were both beneficial in the subgroup analysis. Further analysis of treatment duration did not indicate that treatment outcomes change with a long course of treatment (e.g., more than 4 weeks). The safety of acupuncture should be further validated, because most of the included studies did not mention adverse events. In addition, the overall “low” GRADE assessment results made our certainty in recommending this therapy to clinical practice as “low.”

Manual and electro-acupuncture combined with routine rehabilitation improved FMA more than rehabilitation alone did. Moreover, the results reached the minimal clinical important difference (MCID) suggested by Page et al. (55) and Chen et al. (56) (5.2 and 4.58, respectively). Our results, based on 29 RCTs, confirmed the results of one previous systematic review (12) that evaluated FMA in six RCTs. Another previously published review (14) showed no benefit of acupuncture, however, the review only included two manual acupuncture RCTs in its meta-analysis. In contrast, our review included more studies with a larger sample size. It should also be noted that recent studies have sought to improve their methodology quality, with half of the included studies reporting appropriate randomization sequence generation. These will provide a greater certainty of the evidence of acupuncture's effect on motor function.

In terms of pain VAS, our review showed acupuncture combined with routine rehabilitation was much more effective than rehabilitation alone at reducing pain. These results are consistent with a recent systematic review of acupuncture for post-stroke shoulder pain (57). Furthermore, our review showed that manual and electro-acupuncture both help to relieve pain when used in combination with routine rehabilitation. This could reinforce the evidence for acupuncture in reducing pain associated with post-stroke SHS (Table 2).

The mechanism of how acupuncture relieves pain has been extensively studied. Electro-acupuncture alleviates sensory and affective inflammatory pain by acting through bioactive chemicals, including opioids, serotonin, and norepinephrine; glutamate receptors and transporters; cytokines; and signal molecules (58–63). With regard to how acupuncture helps to improve motor function, experimental studies indicate it may work by enhancing the gamma-aminobutyric acid receptor expression or promoting angiogenesis (64, 65). Currently, there is no consensus on the mechanism of acupuncture for SHS or CRPS, so further research is needed to investigate how acupuncture influences sympathetic/somatic nervous system dysfunction.

### Study Strengths and Limitations

Our systematic review has several strengths. First, we explored the effects of acupuncture for a specific complication (SHS) after stroke, rather than general motor function. Acupuncture points around the shoulder were the most frequently points used in treatment for SHS, which is consistent with two previous reviews (57, 66). These results may provide more focused evidence to improve clinical practice and health outcomes. Second, this systematic review was based on a comprehensive and up-to-date search of literature, with reliable evidence generated from analyses with large sample sizes. Third, subgroup analyses were conducted according to the type of acupuncture stimulation. Both electro-acupuncture and manual acupuncture were effective when they were used as add-on therapies. Fourth, validated outcome measures were selected to assess acupuncture's efficacy, which will also strengthen the reliability of our results.

However, some limitations of this review should be considered before its results can be translated to clinical practice. First, none of the studies used sham or placebo acupuncture in the control treatment. This means that the placebo effects of adding an intervention cannot be ruled out. In particular, Chinese populations usually have high expectations for acupuncture and this may have inflated treatment outcomes for the combined therapy. Second, the treatment duration and number of treatment sessions in the included studies varied greatly. Although we conducted a subgroup analysis based on treatment duration, the optimal duration, and number of treatment sessions are still unclear. Third, the included studies did not give enough information about the acupuncture parameters, such as depth of needle insertion and the acupuncturists' backgrounds. This may affect the clinical implications of this research. Fourth, few studies reported adverse events, making it difficult for us to provide a conclusion about acupuncture's safe use in the management of post-stroke SHS. Moreover, none of the studies performed a long-term follow-up investigation of acupuncture, so it's unclear if acupuncture has a sustained effect on post-stroke SHS. Future clinical studies should include a sham/placebo control, a follow-up phase, and an in-depth data collection of adverse events.

### Implication for Further Research

All of the studies included in this review were conducted on mainland China. Further studies should be done around the world to involve more ethnically and culturally diverse populations. The methodological quality of future clinical trials should also be improved. In particular, double-blinding should be used where possible to avoid RCT performance bias. While it's difficult to have genuine double-blinding in acupuncture trials because acupuncturists have to be aware of the participants' group allocation to treat them, participants can be blinded by using well-designed placebo acupuncture devices and sham acupuncture (67, 68). Outcome assessors can also be blinded. Finally, reports from future RCTs should follow the Consolidated Standards of Reporting Trials (CONSORT) statement and STRICTA checklists (69, 70).

## Conclusion

This systematic review shows that adding acupuncture to routine rehabilitation can improve clinical outcomes (pain and motor function) for people with mild post-stroke SHS. However, the evidence was assessed as “low” by GRADE due to the methodological limitations and heterogeneity of included studies, which made our certainty in recommending acupuncture for this condition in clinical practice as “low.” Well-designed placebo-controlled RCTs with a long treatment duration and follow up, as well as standardized reporting, are needed to support acupuncture's safe and effective use in the management of post-stroke SHS.

## Author Contributions

SL and CZ designed this study. SL, CZ, and YC performed data extraction and statistical analysis. SL, CZ, YC, XG, AZ, CX, and CL revised and approved the final manuscript.

### Conflict of Interest Statement

The authors declare that the research was conducted in the absence of any commercial or financial relationships that could be construed as a potential conflict of interest.

## Supplementary Material

Figure S1 The summary of risk of bias graph.

Appendix S1 Search strategy.

## References

[1] Kocabas H Levendoglu F Ozerbil OM Yuruten B. Complex regional pain syndrome in stroke patients. Int J Rehabil Res. (2007) 30:33–8. 10.1097/MRR.0b013e3280146f57 17293718

[2] Petchkrua W Weiss DJ Patel RR. Reassessment of the incidence of complex regional pain syndrome type 1 following stroke. Neurorehabil Neural Repair. (2000) 14:59–63. 10.1177/154596830001400107 11228950

[3] Pertoldi S Di Benedetto P. Shoulder-hand syndrome after stroke. A complex regional pain syndrome. Eura Medicophys. (2005) 41:283–92. 16474282

[4] Swati M Teasull R Foley N Evidence-Based Review of Stroke Rehabilitation–Hemiplegic Shoulder Pain. Available online at: www.ebrsr.com (accessed April 10, 2018).

[5] Forouzanfar T Koke AJ van Kleef M Weber WE. Treatment of complex regional pain syndrome type I. Eur J Pain. (2002) 6:105–22. 10.1053/eujp.2001.0304 11900471

[6] Hu HH Chung C Liu TJ Chen RC Chen CH Chou P. A randomized controlled trial on the treatment for acute partial ischemic stroke with acupuncture. Neuroepidemiology. (1993) 12:106–13. 10.1159/000110308 8232703

[7] Wu P Mills E Moher D Seely D. Acupuncture in poststroke rehabilitation: a systematic review and meta-analysis of randomized trials. Stroke. (2010) 41:e171–9. 10.1161/STROKEAHA.109.573576 20167912

[8] Park J Hopwood V White AR Ernst E. Effectiveness of acupuncture for stroke: a systematic review. J Neurol. (2001) 248:558–63. 10.1007/s004150170132 11517996

[9] Sze FK Wong E Or KK Lau J Woo J. Does acupuncture improve motor recovery after stroke? A meta-analysis of randomized controlled trials. Stroke. (2002) 33:2604–19. 10.1161/01.STR.0000035908.74261.C9 12411650

[10] Yang A Wu HM Tang JL Xu L Yang M Liu GJ Acupuncture for stroke rehabilitation. Cochrane Database Syst Rev. (2016) 8:CD004131 10.1002/14651858.CD004131.pub3 PMC6464684 27562656

[11] Zhang SH Liu M Asplund K Li L Acupuncture for acute stroke. Cochrane Database Syst Rev. (2005) 2:CD003317 10.1002/14651858.CD003317.pub2 15846657

[12] Xu Y Li W Liu J Ma L Acupuncture versus rehabilitation therapy for shoulder hand syndrome: a systematic review and meta-analysis [Article in Chinese]. Lishizhen Med Mater Med Res. (2013) 24:1794–8. 10.3969/j.issn.1008-0805.2013.07.114

[13] Lu Y Fu L Mu J Xu H Qi Y Acupuncture for post stroke shoulder hand syndrome: a systematic review [Article in Chinese]. Chinese J Evid-based Med. (2009) 9:976–8. 10.3969/j.issn.1672-2531.2009.09.010

[14] Liu K Han X Pan X Xiong J Acupuncture in the treatment of poststroke shoulder hand syndrome: a systematic review [Article in Chinese]. Chinese J Rehabil Med. (2015) 30:1041–5. 10.3969/j.issn.1001-1242.2015.10.014

[15] Savovic J Jones HE Altman DG Harris RJ Juni P Pildal J. Influence of reported study design characteristics on intervention effect estimates from randomized, controlled trials. Ann Intern Med. (2012) 157:429–38. 10.7326/0003-4819-157-6-201209180-00537 22945832

[16] Gao P Li Y Wang Z Jiang M Luo Y Effect of electro-acupuncture combined with rehabilitation for the treatment of shoulder hand syndrome [Article in Chinese]. Chin Manipul Rehabilit Med. (2011) 2:49–50.

[17] Duan Y Liu F Zhang Y Effect of electro-acpuncture combined with rehabilitation training on shoulder hand syndrome [Article in Chinese]. Jilin J Trad Chin Med. (2014) 34:190–1. 10.13463/j.cnki.jlzyy.2014.02.031

[18] Li M Clinical research of electro-acupuncture on Large intestine meridian five acupoint for the treatment of poststroke should-hand syndrome [Article in Chinese]. Jilin J Trad Chin Med. (2013) 33:191–2. 10.3969/j.issn.1003-5699.2013.02.041

[19] Xu S Zhuang L Jia C Xu D Pan C Clinical observation of Jin's Three-needle manipulation combined with rehabilitation therapy for treatment of post-stroke shoulder-hand syndrome [Article in Chinese]. J Guangzhou University Trad Chin Med. (2010) 27:19–22. 10.13359/j.cnki.gzxbtcm.2010.01.009

[20] Hou Z Effect of acupuncture combined with rehabilitation on stage I of shoulder-hand syndrome after stroke [Article in Chinese]. J Clin Med in Prac. (2014) 18:58–59, 66.

[21] Jia C Ni G Tan H Zhang X Clinical effect of acupuncture combined with rehabilitation on stage I of shoulder-hand syndrome after stroke [Article in Chinese]. J Changchun University Trad Chin Med. (2012) 28:711–2. 10.13463/j.cnki.cczyy.2012.04.084

[22] Zhou F Zeng K Lu Y Clincial effect of Taichi acupuncture combined with rehabilitation for the treatment of poststroke shoulder-hand syndrome [Article in Chinese]. Modern Diagn Treatment. (2013) 24:1004–6. 10.3969/j.issn.1001-8174.2013.05.027

[23] Liu Y Clinical research of treating shoulder-hand syndrome by contralateral acupuncture on shoulder pain point combining with rehabilitation training [Article in Chinese]. Gansu Med J. (2009) 28:176–8. 10.15975/j.cnki.gsyy.2009.03.002

[24] Su J Pan C Wan X The balancing acupuncture combined with rehabilitation for the treatment of shoulder-hand syndrome after stroke [Article in Chinese]. Chin J Rehab. (2010) 25:188–9. 10.3870/zgkf.2010.03.010

[25] Wan X Su J Ye Z Luo R Zeng Y Effects of balancing acupuncture combined with rehabilitation in treatment of shoulder-hand syndrome after stroke [Article in Chinese]. Int Med Health Guid News. (2012) 18:3534–7. 10.3760/cma.j.issn.1007-1245.2012.24.002

[26] Yin J Zhou G Zhou G Fan H Therapeutic observation of acupuncture at the interiorly-exteriorly related meridians plus rehabilitation training for poststroke shoulder-hand syndrome [Article in Chinese]. Shanghai J Acupunct Moxibust. (2015) 34:7–10. 10.13460/j.issn.1005-0957.2015.01.0007

[27] Qin H Shi J Zhang Y Ma D Gao Q Observations on the efficacy of scalp point-to-point acupuncture plus rehabilitation training in treating poststroke shoulder-hand syndrome [Article in Chinese]. Shanghai J Acupunct Moxibust. (2013) 32:167–169. 10.3969/j.issn.1005-0957.2013.03.167

[28] Shang Y Ma C Cai Y Wang D Kong L. Clinical study on acupuncture combined with rehabilitation therapy for treatment of poststroke shoudler-hand syndrome [Article in Chinese]. Chinese J Acupunct Moxibust. (2008) 28:331–3. 10.13703/j.0255-2930.2008.05.017 18652322

[29] Wan W Wang T Cheng S Zhao Y Zhang W Wu Q Poststroke shoulder-hand syndrome treated with acupuncture and rehabilitation: a randomized controlled trial [Article in Chinese]. Chinese J Acupunct Moxibust. (2013) 33:970–4. 10.13703/j.0255-2930.2013.11.026 24494280

[30] Lin H Ye G Liao H Lin F Liang B Acupuncture combined with rehabilitation training in the treatment of shoulder-hand syndrome after stroke [Article in Chinese]. World Chin Med. (2014) 9:84–85, 88. 10.3969/j.issn.1673-7202.2014.01.030

[31] Wang X Gao C Ma S Clinica study on acupuncture combined with rehabilitation training in treatment of shoulder-hand syndrome after stroke [Article in Chinese]. J Clin Exp Med. (2012) 11:942–3. 10.3969/j.issn.1671-4695.2012.12.018

[32] Chen M Clinical observation on the resluts of treatment of shoulder-hand syndrome due to cerebral stroke with acupuncture [Article in Chinese]. China Trop Med. (2008) 8:1781, 1717. 10.3969/j.issn.1009-9727.2008.10.061

[33] Liao H Clinical observation on the efficacy of occupational therapy plus acupuncture for treating reflex sympathetic dystrophy [Article in Chinese]. Shanghai J Acupunct Moxibust. (2006) 25:9–10. 10.3969/j.issn.1005-0957.2006.03.004

[34] Li F Tu M Effect of electroacupuncture combined with occupational therapy on stage I of shoulder-hand syndrome after stroke [Article in Chinese]. China J Pharmaceut Econom. (2013) :370–1.

[35] Zheng S Wu Y Chang J Cui S Xu M Lian J Clinical effect of acupuncture on shoulder-hand syndrome after stroke [Article in Chinese]. Chinese J Rehabil Med. (2013) 28:40–1. 10.3870/zgkf.2013.01.015

[36] Xu Z Clinical Observation of Electro-Acupuncture on Large Intestine Meridian Five Acupoint for the Treatment of Poststroke Should-Hand Syndrome [Dissertation]. Changchun, Changchun University of Chinese Medicine (2008).

[37] You Y A Clinical Study of Electroacupuncture Combined with Rehabilitation Training in the Treatment of Shoulder-Hand Syndrome of Poststroke [Dissertation]. Jinan, Shandong University of Chinese Medicine (2011).

[38] Zhang X Clinical Research of Acupuncture Combined With Rehabilitation Training for the Treatment of Shoulder-Hand Syndrome after Stroke [Dissertation]. Guangzhou, Guangzhou University of Chinese Medicine (2011).

[39] Wu Z Investigate the Clincial Effect of the Neck Clip Ridge and Through Thorn Acupuncture Methods on Treating the Shoulder-Hand Syndrome [Dissertation]. Harbin, Heilongjiang University of Chinese Medicine (2011).

[40] Xie Q A Clinical Study of Acupuncture Combined with Rehabilitation Training in the Treatment of Shoulder-Hand Syndrome after Stroke [Dissertation]. Guangzhou, Guangzhou University of Chinese Medicine (2010).

[41] Zhong C Ni D Lin W Chen F Clinical observation of acupuncture combined with rehabilitation training for the treatment of poststroke shoulder-hand syndrome [Article in Chinese]. Hainan Med J. (2016) 27:1687–8. 10.3969/j.issn.1003-6350.2016.10.048

[42] Zhao J Clinical Effect of Acupuncture Combined with TDP Lamp Irradiation for the Treatment of Shoulder-Hand Syndrome After Stroke [Dissertation]. Shenyang, Liaoning University of Traditional Chinese Medicine (2016).

[43] Yang D He X Cai W Yang X Yang M Clinical effect of floating acupuncture combined with rehabilitation for the treatment of shoulder-hand syndrome after stroke [Article in Chinese]. Lishizhen Med Mater Med Res. (2015) 26:139–1341.

[44] Xu F Li H Zhang Q Acupuncture combined with rehabilitation for the treatment of poststroke shoulder-hand syndrome: a randomised controlled trial [Article in Chinese]. Chin J Trauma Disability Med. (2015) 3:141–2. 10.13214/j.cnki.cjotadm.2015.16.107

[45] Tang D Wu W Sun X Acupuncture combined with rehabilitation for the treatment of poststroke shoulder-hand syndrome: a randomised controlled trial [Article in Chinese]. J Clin Acupunct Moxibust. (2016) 32:26–9.

[46] Liang N Liu X Clinical observation of electroacupuncture combined with rehabilitation training for the treatment of poststroke shoudler-hand syndrome [Article in Chinese]. Guide China Med. (2016) 14:200–1. 10.15912/j.cnki.gocm.2016.25.163

[47] Li Z Gu J Hu M Clinical observation of acupuncture for the treatment of 46 case of shoudler-hand syndrome after stroke [Article in Chinese]. Chin J Ethnomed Ethnoph. (2015) 24:78–9.

[48] Huang C Fan W Yu A Cui X Wu J Penetration acupuncture at Baxie(EX-UE 9) combinded with rehabilitation for swelling hand of poststroke shoudler-hand syndrome [Article in Chinese]. Chinese J Acupunct Moxibust. (2017) 37:121–124. 10.13703/j.0255-2930.2017.02.004 29231471

[49] He S Gao S Evaluation of abdominal acupuncture and rehabilitation treatment for shoudler-hand syndrome. (period I) after stroke [Article in Chinese]. J Clin Acupunct Moxibust. (2016) 32:11–13.

[50] Feng Y Ma S Acupuncture combined with infrared acupoint irradiation and rehabilitation treatment of 126 cases of clinical observation on shoulder-hand syndrome after stroke [Article in Chinese]. Laser J. (2015) 36:195–7. 10.14016/j.cnki.jgzz.2015.04.195

[51] Chen Y Huang T Liu K Clinical research of using acupuncture and rehabilitation training in the treatment of poststroke shoudler-hand syndrome stage I [Article in Chinese]. Sichuan J Trad Chin Med. (2015) 23:150–2.

[52] Liu Y Zhang H Effect of acupuncture combined with rehabilitation training on patients with acute shoulder-hand syndrome after stroke [Article in Chinese]. J Hun Univers Chin Med. (2018) 38:546–9. 10.3969/j.issn.1674-070X.2018.05.015

[53] Wang X Gao Y Gao S Effect of acupuncture combined with rehabilitation training on PRI, FMA and MBI in patients with shoulder-hand syndrome after stroke [Article in Chinese]. Global Trad Chin Med. (2017) 10:361–3. 10.3969/j.issn.1674-1749.2017.03.033

[54] Schwartzman RJ McLellan TL. Reflex sympathetic dystrophy. A review. Arch Neurol. (1987) 44:555–61. 10.1001/archneur.1987.00520170081028 3495254

[55] Page SJ Fulk GD Boyne P. Clinically important differences for the upper-extremity Fugl-Meyer Scale in people with minimal to moderate impairment due to chronic stroke. Phys Ther. (2012) 92:791–8. 10.2522/ptj.20110009 22282773

[56] Chen R Wu J Shen X A research on the minimal clinically important differences of Chinese version of the Fugl-Meyer motor scale [Article in Chinese]. Acta Univers Med Anhui. (2015) 50:519–22. 10.19405/j.cnki.issn1000-1492.2015.04.025

[57] Lee JA Park SW Hwang PW Lim SM Kook S Choi KI. Acupuncture for shoulder pain after stroke: a systematic review. J Altern Complement Med. (2012) 18:818–23. 10.1089/acm.2011.0457 22924414 PMC3429280

[58] Zhang R Lao L Ren K Berman BM. Mechanisms of acupuncture-electroacupuncture on persistent pain. Anesthesiology. (2014) 120:482–503. 10.1097/ALN.0000000000000101 24322588 PMC3947586

[59] Zhang GG Yu C Lee W Lao L Ren K Berman BM. Involvement of peripheral opioid mechanisms in electroacupuncture analgesia. Explore. (2005) 1:365–71. 10.1016/j.explore.2005.06.006 16781567

[60] Li A Zhang RX Wang Y Zhang H Ren K Berman BM. Corticosterone mediates electroacupuncture-produced anti-edema in a rat model of inflammation. BMC Complement Altern Med. (2007) 7:27. 10.1186/1472-6882-7-27 17697336 PMC1976320

[61] Lee JH Jang KJ Lee YT Choi YH Choi BT. Electroacupuncture inhibits inflammatory edema and hyperalgesia through regulation of cyclooxygenase synthesis in both peripheral and central nociceptive sites. Am J Chin Med. (2006) 34:981–8. 10.1142/S0192415X06004454 17163587

[62] Sekido R Ishimaru K Sakita M. Corticotropin-releasing factor and interleukin-1beta are involved in the electroacupuncture-induced analgesic effect on inflammatory pain elicited by carrageenan. Am J Chin Med. (2004) 32:269–79. 10.1142/S0192415X04001928 15315264

[63] Goldman N Chen M Fujita T Xu Q Peng W Liu W. Adenosine A1 receptors mediate local anti-nociceptive effects of acupuncture. Nat Neurosci. (2010) 13:883–8. 10.1038/nn.2562 20512135 PMC3467968

[64] Xu Q Yang JW Cao Y Zhang LW Zeng XH Li F. Acupuncture improves locomotor function by enhancing GABA receptor expression in transient focal cerebral ischemia rats. Neurosci Lett. (2015) 588:88–94. 10.1016/j.neulet.2014.12.057 25556683

[65] Du Y Shi L Li J Xiong J Li B Fan X. Angiogenesis and improved cerebral blood flow in the ischemic boundary area were detected after electroacupuncture treatment to rats with ischemic stroke. Neuro Res. (2011) 33:101–7. 10.1179/016164110X12714125204317 20546685

[66] Lin Y Li Z Fu J Liu X The systematic evaluation of acupuncture combined with rehabilitation training for the treatment of poststroke shoudler pain [Article in Chinese]. J Nurses Train. (2015) 30:1004–9. 10.16821/j.cnki.hsjx.2015.11.011

[67] Zhang CS Tan HY Zhang GS Zhang AL Xue CC Xie YM. Placebo devices as effective control methods in acupuncture clinical trials: a systematic review. PLoS ONE. (2015) 10:e0140825. 10.1371/journal.pone.0140825 26536619 PMC4633221

[68] Moroz A Freed B Tiedemann L Bang H Howell M Park JJ. Blinding measured: a systematic review of randomized controlled trials of acupuncture. Evid Based Complement Alternat Med. (2013) 2013:708251. 10.1155/2013/708251 23533515 PMC3603669

[69] Moher D Hopewell S Schulz KF Montori V Gotzsche PC Devereaux PJ. CONSORT 2010 explanation and elaboration: updated guidelines for reporting parallel group randomised trials. BMJ. (2010) 340:c869. 10.1136/bmj.c869 20332511 PMC2844943

[70] MacPherson H Altman DG Hammerschlag R Youping L Taixiang W White A. Revised Standards for Reporting Interventions in Clinical Trials of Acupuncture. (STRICTA): extending the CONSORT statement. PLoS Med. (2010) 7:e1000261. 10.1371/journal.pmed.1000261 20543992 PMC2882429

