# Supplementary figures and images for: Acupuncture for Post-stroke Shoulder-Hand Syndrome: A Systematic Review and Meta-Analysis
Source: Front Neurol. 2019 Apr 26;10:433. doi: 10.3389/fneur.2019.00433 (PMC6498454; doi:10.3389/fneur.2019.00433)

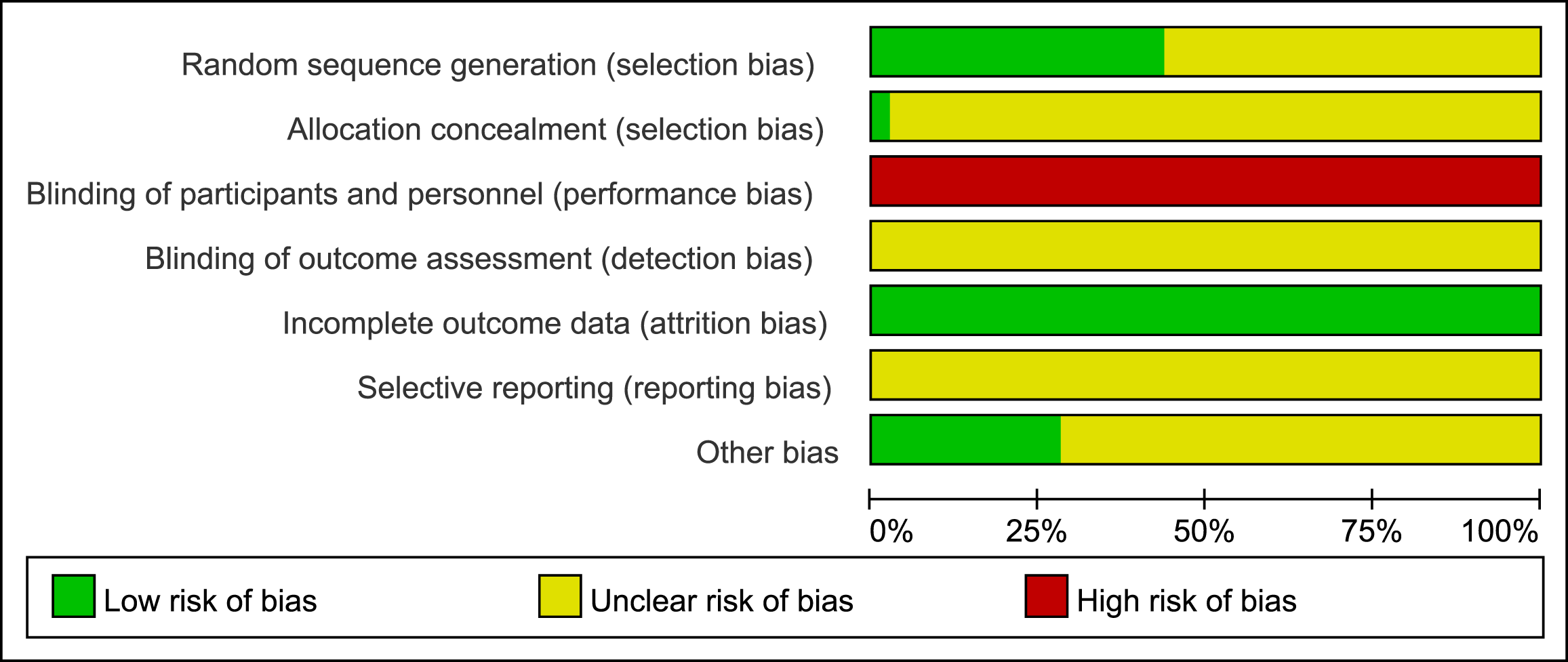

Supplement: Figure S1 — The summary of risk of bias graph. [file Image_1.TIF]
