# Supplementary material for: Acupuncture for Post-stroke Shoulder-Hand Syndrome: A Systematic Review and Meta-Analysis
Source: Front Neurol. 2019 Apr 26;10:433. doi: 10.3389/fneur.2019.00433 (PMC6498454; doi:10.3389/fneur.2019.00433)
Supplement: Appendix S1 — Search strategy. [file Table_1.DOCX]

Appendix S1 Search strategy

| No. | Search strategy |
| --- | --- |
| #1 | ((((((("stroke"[MeSH Terms] OR "stroke"[All Fields]) OR ("brain infarction"[MeSH Terms] OR ("brain"[All Fields] AND "infarction"[All Fields]) OR "brain infarction"[All Fields])) OR ("intracranial embolism and thrombosis"[MeSH Terms] OR ("intracranial"[All Fields] AND "embolism"[All Fields] AND "thrombosis"[All Fields]) OR "intracranial embolism and thrombosis"[All Fields])) OR ("intracranial haemorrhages"[All Fields] OR "intracranial hemorrhages"[MeSH Terms] OR ("intracranial"[All Fields] AND "hemorrhages"[All Fields]) OR "intracranial hemorrhages"[All Fields])) OR ("stroke"[MeSH Terms] OR "stroke"[All Fields] OR ("cerebrovascular"[All Fields] AND "accident"[All Fields]) OR "cerebrovascular accident"[All Fields])) OR ("cerebral infarction"[MeSH Terms] OR ("cerebral"[All Fields] AND "infarction"[All Fields]) OR "cerebral infarction"[All Fields])) OR ("intracranial haemorrhage"[All Fields] OR "intracranial hemorrhages"[MeSH Terms] OR ("intracranial"[All Fields] AND "hemorrhages"[All Fields]) OR "intracranial hemorrhages"[All Fields] OR ("intracranial"[All Fields] AND "hemorrhage"[All Fields]) OR "intracranial hemorrhage"[All Fields])) OR ("cerebral haemorrhage"[All Fields] OR "cerebral hemorrhage"[MeSH Terms] OR ("cerebral"[All Fields] AND "hemorrhage"[All Fields]) OR "cerebral hemorrhage"[All Fields]) |
| #2 | ("acupuncture"[MeSH Terms] OR "acupuncture"[All Fields] OR "acupuncture therapy"[MeSH Terms] OR ("acupuncture"[All Fields] AND "therapy"[All Fields]) OR "acupuncture therapy"[All Fields]) OR ("meridians"[MeSH Terms] OR "meridians"[All Fields]) OR ("electroacupuncture"[MeSH Terms] OR "electroacupuncture"[All Fields]) OR ("moxibustion"[MeSH Terms] OR "moxibustion"[All Fields]) OR ("auriculotherapy"[MeSH Terms] OR "auriculotherapy"[All Fields]) OR (("prunus domestica"[MeSH Terms] OR ("prunus"[All Fields] AND "domestica"[All Fields]) OR "prunus domestica"[All Fields] OR "plum"[All Fields]) AND ("flowers"[MeSH Terms] OR "flowers"[All Fields] OR "blossom"[All Fields])) OR ("acupressure"[MeSH Terms] OR "acupressure"[All Fields]) OR ("acupuncture, ear"[MeSH Terms] OR ("acupuncture"[All Fields] AND "ear"[All Fields]) OR "ear acupuncture"[All Fields] OR ("ear"[All Fields] AND "acupuncture"[All Fields])) OR (("ear"[MeSH Terms] OR "ear"[All Fields]) AND ("acupressure"[MeSH Terms] OR "acupressure"[All Fields])) OR ("acupuncture, ear"[MeSH Terms] OR ("acupuncture"[All Fields] AND "ear"[All Fields]) OR "ear acupuncture"[All Fields] OR ("acupuncture"[All Fields] AND "ear"[All Fields]) OR "acupuncture, ear"[All Fields]) OR ("acupuncture therapy"[MeSH Terms] OR ("acupuncture"[All Fields] AND "therapy"[All Fields]) OR "acupuncture therapy"[All Fields]) OR moxa[All Fields] OR (("lasers"[MeSH Terms] OR "lasers"[All Fields] OR "laser"[All Fields]) AND ("acupuncture"[MeSH Terms] OR "acupuncture"[All Fields] OR "acupuncture therapy"[MeSH Terms] OR ("acupuncture"[All Fields] AND "therapy"[All Fields]) OR "acupuncture therapy"[All Fields])) OR (seven[All Fields] AND ("Star"[Journal] OR "star"[All Fields]) AND ("needles"[MeSH Terms] OR "needles"[All Fields] OR "needle"[All Fields])) OR ("acupuncture analgesia"[MeSH Terms] OR ("acupuncture"[All Fields] AND "analgesia"[All Fields]) OR "acupuncture analgesia"[All Fields]) OR ("acupuncture points"[MeSH Terms] OR ("acupuncture"[All Fields] AND "points"[All Fields]) OR "acupuncture points"[All Fields]) OR electro-acupuncture[All Fields] OR (electro[All Fields] AND ("acupuncture"[MeSH Terms] OR "acupuncture"[All Fields] OR "acupuncture therapy"[MeSH Terms] OR ("acupuncture"[All Fields] AND "therapy"[All Fields]) OR "acupuncture therapy"[All Fields])) OR ("transcutaneous electric nerve stimulation"[MeSH Terms] OR ("transcutaneous"[All Fields] AND "electric"[All Fields] AND "nerve"[All Fields] AND "stimulation"[All Fields]) OR "transcutaneous electric nerve stimulation"[All Fields] OR "tens"[All Fields]) OR ("transcutaneous electric nerve stimulation"[MeSH Terms] OR ("transcutaneous"[All Fields] AND "electric"[All Fields] AND "nerve"[All Fields] AND "stimulation"[All Fields]) OR "transcutaneous electric nerve stimulation"[All Fields] OR ("transcutaneous"[All Fields] AND "nerve"[All Fields] AND "stimulation"[All Fields]) OR "transcutaneous nerve stimulation"[All Fields]) OR ("transcutaneous electric nerve stimulation"[MeSH Terms] OR ("transcutaneous"[All Fields] AND "electric"[All Fields] AND "nerve"[All Fields] AND "stimulation"[All Fields]) OR "transcutaneous electric nerve stimulation"[All Fields]) OR ("transcutaneous electric nerve stimulation"[MeSH Terms] OR ("transcutaneous"[All Fields] AND "electric"[All Fields] AND "nerve"[All Fields] AND "stimulation"[All Fields]) OR "transcutaneous electric nerve stimulation"[All Fields] OR ("transcutaneous"[All Fields] AND "electrical"[All Fields] AND "nerve"[All Fields] AND "stimulation"[All Fields]) OR "transcutaneous electrical nerve stimulation"[All Fields]) OR electro-stimulation[All Fields] OR (electro[All Fields] AND stimulation[All Fields]) OR pharmacopuncture[All Fields] OR (point[All Fields] AND ("injections"[MeSH Terms] OR "injections"[All Fields] OR "injection"[All Fields])) OR (("catgut"[MeSH Terms] OR "catgut"[All Fields]) AND embedding[All Fields]) |
| #3 | "randomized controlled trial"[pt] OR "controlled clinical trial"[pt] OR "randomized"[tiab] OR "placebo"[tiab] OR "drug therapy"[sh] OR "randomly"[tiab] OR "trial"[tiab] OR "groups"[tiab] |
| #4 | #1 AND #2 AND #3 |
